# Let's talk emotions: from classification and early intervention to suicide prevention

**DOI:** 10.1192/bji.2024.22

**Published:** 2024-11

**Authors:** Marinos Kyriakopoulos

**Affiliations:** Assistant Professor in Child and Adolescent Psychiatry, School of Medicine, National and Kapodistrian University of Athens, Athens, Greece. Email: mkyriakop@med.uoa.gr

Understanding, defining, describing and managing emotions are among the most challenging aspects of mental health work and a core element of psychiatric input across the world. Emotional disorders may bring a heavy burden of suffering and are associated with significant morbidity, excess mortality and with stigma and shame. Several important questions about their nosological status, effective early intervention and prevention of adverse outcomes, and broader public health strategies mitigating their risks, remain to a large extent unanswered.

In this issue of *BJPsych International*, we have three articles addressing some aspects of these conditions: two focusing on borderline personality disorder (BPD)^1^ and emotional dysregulation with maladaptive coping^2^ and the third discussing suicide.^3^ In the first of these articles, Jay Watts^1^ argues that the construct of BPD is unsatisfactory, heterogeneous and overlaps with many other conditions, and that the concept of epistemic injustice may be used to understand power dynamics associated with its ongoing use. It is proposed that more affirmative alternative diagnoses, not locating the problem in the person's own being, would be less harmful and better describe the struggles a person has without hindering access to transdiagnostic evidence-based treatments.

In the second paper, Khan and colleagues,^2^ focusing on early intervention, present a pioneering pilot implementation of group dialectical behaviour therapy for adolescents with emotional dysregulation and maladaptive coping in Qatar's child and adolescent mental health services. The study highlights the positive impact of this approach on patient satisfaction and the potential for early intervention for this indication in the Middle Eastern and North Africa region. Importantly, stigma, accessibility and cultural needs were addressed through family involvement and community support in line with cultural expectations.

Finally, Riaz and colleagues^3^ use the rise in self-harm and suicide in Balochistan, Pakistan's largest province, to reflect on the need for suicide prevention strategies through public health initiatives, community-based mental health services, education and research. They suggest establishing a suicide prevention task force, inclusion of life skills and mental health education in school and undergraduate curricula, conducting research into self-harm, fostering empathetic media reporting, and promoting sustainable economic change.

Considering emotional disorders within environmental, developmental and cultural backgrounds is likely to further advance effective interventions and strategies and prevent adverse outcomes. Adaptations of our approach related to context may help reduce their global impact.

## Data Availability

Data availability is not applicable to this article as no new data were created or analysed in this work.
